# Associations between the TyG index and the ɑ-Klotho protein in middle-aged and older population relevant to diabetes mellitus in NHANES 2007–2016

**DOI:** 10.1186/s12944-024-02172-3

**Published:** 2024-06-21

**Authors:** Shujuan Qiu, Chunlei Li, Jinhua Zhu, Zhentao Guo

**Affiliations:** 1 Department of Nephrology, Affiliated Hospital of Shandong Second Medical University, No. 2428, Yuhe Road, Quiwen District, Weifang, 261041 Shandong China; 2 Zhucheng Nanhu Community Health Service Center, No. 2000, Tourism Road, South Lake Ecological Economic Development District, Zhucheng, 262200 Shandong China

**Keywords:** TyG, Soluble α-Klotho, Diabetes, Sex, Cross-sectional study

## Abstract

**Background:**

The anti-aging protein Klotho has diverse functions in antioxidative stress and energy metabolism through several pathways. While it has been reported that α-Klotho is downregulated in patients with insulin resistance (IR), the association between Klotho and IR is complex and controversial. The triglyceride-glucose (TyG) index has provided a practical method for assessing IR. With this in mind, our study aimed to investigate the relationship between the TyG index and soluble α-Klotho protein levels in US populations, both with and without diabetes mellitus.

**Methods:**

This cross-sectional study analyzed data from middle-aged and older participants in the National Health and Nutrition Examination Survey (NHANES) conducted between 2007 and 2016. The participants were divided into two groups based on their diabetes mellitus status: those with diabetes and those without diabetes. To evaluate the relationship between the TyG index and the concentration of the α-Klotho protein in each group, a series of survey-weighted multivariable linear regression models were employed. Furthermore, to examine the association between these two variables, multivariable-adjusted restricted cubic spline curves and subgroup analysis were generated.

**Results:**

The study involved 6,439 adults aged 40 years or older, with a mean age of 57.8 ± 10.9 years. Among them, 1577 (24.5%) had diabetes mellitus. A subgroup analysis indicated that the presence of diabetes significantly affected the relationship between the TyG index and the α-Klotho level. After considering all covariables, regression analysis of the participants without diabetes revealed that the α-Klotho concentration decreased by 32.35 pg/ml (95% CI: -50.07, -14.64) with each one unit increase in TyG (*p* < 0.001). The decline in α-Klotho levels with elevated TyG was more pronounced in the female population. In patients with diabetes mellitus, a non-linear association between the TyG index and α-Klotho was observed. There was no significant correlation observed between the two when TyG index were below 9.7. However, there was an increase in klotho levels of 106.44 pg/ml for each unit increase in TyG index above 9.7 (95% CI: 28.13, 184.74) (*p* = 0.008).

**Conclusion:**

Our findings suggested that the presence of diabetes may influence the relationship between the TyG index and soluble α-Klotho. Furthermore, there seem to be sex differences in individuals without diabetes. Further studies are necessary to validate these findings.

**Supplementary Information:**

The online version contains supplementary material available at 10.1186/s12944-024-02172-3.

## Introduction

The Klotho gene is intricately linked to lifespan and is implicated in premature aging processes [1]. Human physiology encompasses three distinct forms of Klotho: α-Klotho, which is a full-length transmembrane protein; soluble α-Klotho; and secreted α-Klotho [2]. Although the specific biological mechanism of the Klotho protein has not been elucidated, it is acknowledged for its multifaceted functions. These include the inhibition of insulin/insulin-like growth factor 1 (IGF-1), the regulation of energy and mineral metabolism, and the attenuation of oxidative stress and inflammatory responses [1, 3]. Recent investigations have illuminated the role of Klotho in the pathogenesis of type 2 diabetes and insulin resistance (IR) [3, 4]. Moreover, a study delineated an association between impaired glucose metabolism in obese mice and reduced expression of α-Klotho [5]. Klotho-deficient mice (kl/kl) exhibit reduced pancreatic insulin content and hypoinsulinemia [6]. Prior studies have indicated decreased plasma levels of α-Klotho in individuals with type 2 diabetes and obesity. This finding suggests the potential contribution of decreased Klotho levels to the onset of glucose and lipid metabolism disorders.

The involvement of α-Klotho in metabolic regulation is complex. Analyses of glucose and insulin tolerance suggest that α-Klotho may exert an inhibitory effect on insulin activity [3]. Notably, the overexpression of α-Klotho has been linked to the development of IR [7]. Conversely, the knockout of Klotho in Lep (ob/ob) mice, characterized by leptin deficiency, has been shown to lead to a reduction in obesity and an increase in insulin sensitivity, ultimately resulting in diminished blood sugar levels [5]. The complexity of the role of α-Klotho in metabolic regulation stems from the diverse physiological functions of α-Klotho and its paradoxical impact on insulin activity [8–10].

The TyG index serves as a valuable tool for assessing IR and is calculated as Ln (fasting triglycerides [TG, mg/dl] × fasting blood glucose [mg/dl]/2) [11, 12]. Recent studies have revealed its association with various factors, such as the inflammatory response, oxidative stress, muscle mass, and kidney disease [13–16]. Notably, these are the domains in which klotho assumes a pivotal role [17, 18].

Currently, there is a dearth of research exploring the association between TyG and α-Klotho levels. Nonetheless, it is imperative to evaluate the relationship between glycolipid metabolic status and the TyG index across diverse metabolic states, considering the significant impact of the former on the latter. Consequently, this study aimed to scrutinize the association between the TyG index and α-Klotho levels in distinct cohorts of individuals, both with and without diabetes.

## Subjects and methods

### Study population

The National Health and Nutrition Examination Survey (NHANES) conducted in the United States employs a sampling method that entails the random selection of individuals from the general population. Detailed technical information about the sample design is available on the NHANES Survey Methods and Analytic Guidelines page [19, 20]. Participants underwent extensive interviews, medical and physiological assessments, and laboratory testing of serum, plasma, urine, and DNA. Approval for the study protocol was granted by the National Center for Health Statistics Research Ethics Review Board, and written informed consent was obtained from all participants [21]. The data were collected continuously and published in two-year cycles. The original study protocol can be accessed on the NHANES Ethics Review Committee website (https://www.cdc.gov/nchs/nhanes/irba98.htm) and received formal approval from the Ethics Review Committee (protocol #2005–06; #2011–17). In this study, a secondary analysis of the data was conducted following the Strengthening the Reporting of Observational Studies in Epidemiology (STROBE) guidelines.

**Fig. 1:**
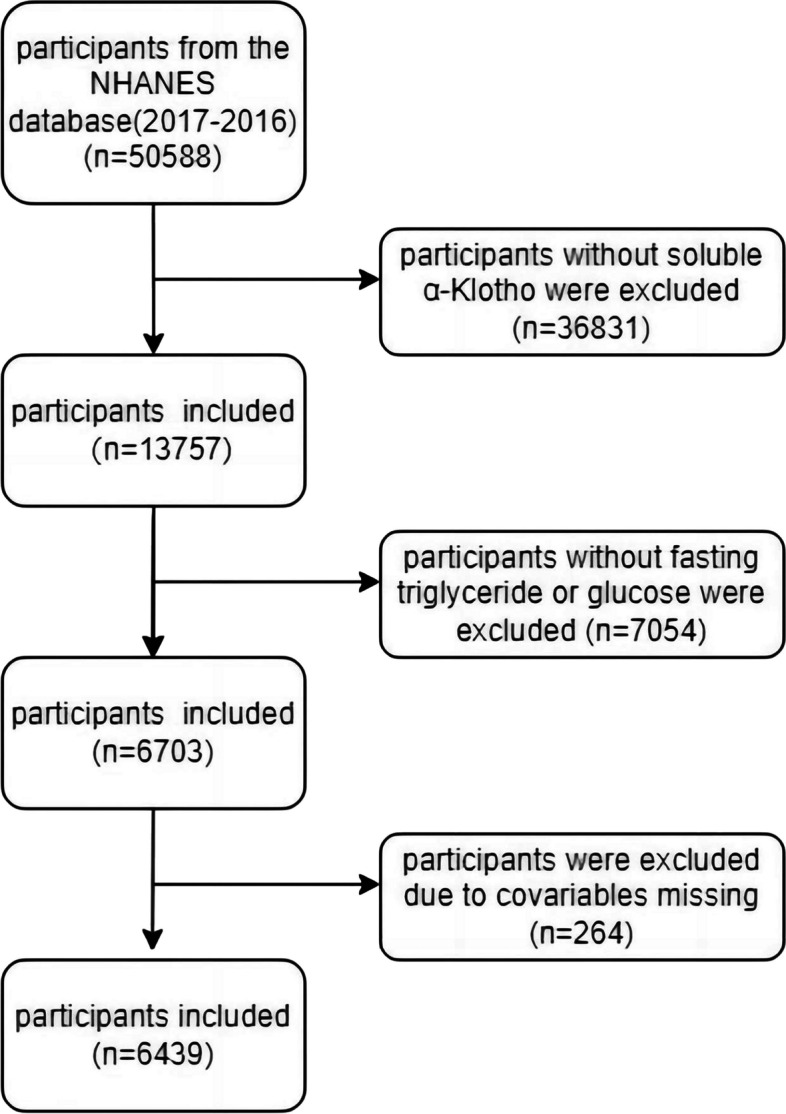
Flowchart of sample selection from the National Health and Nutrition Examination Survey (NHANES) 2007–2016

A total of five NHANES cycles, spanning from 2007 to 2016, were analyzed in our study. In the NHANES study, α-Klotho levels were measured exclusively in individuals aged 40 years and older. Therefore, our study focused on a population of middle-aged and older adults. We specifically recruited adults aged 40 years or older who had undergone measurements of fasting triglycerides (TG), fasting blood glucose (FBG), and soluble α-Klotho. Participants with missing data on soluble α-Klotho, FBG, TG or other relevant factors were excluded. The analysis involved a total of 6439 individuals (refer to Fig. 1). All participants were stratified based on whether they had diabetes or not. Diabetes status was defined based on the following criteria: (1) a documented diagnosis from a healthcare provider, (2) a fasting plasma glucose level ≥ 7.0, (3) a glycosylated hemoglobin (HbA1c) level ≥ 6.5, or (4) the use of medications for diabetes [22].

### Exposure and outcome variables

The TyG index was designated as the independent variable and calculated based on TG and FBG using the formula mentioned earlier [11]. TG and FBG levels were determined through an enzymatic assay conducted on an automated biochemistry analyzer. TG concentrations were measured utilizing a Roche Modular P and Roche Cobas 6000 chemistry analyzers, while FBG concentrations were assessed through hexokinase-mediated reactions on a Roche/Hitachi Cobas C 501 chemistry analyzer.

Our investigation focused on the dependent variable, soluble α-Klotho. As per the NHANES website, serum samples were preserved at -80 °C until they were prepared for analysis. A commercially available enzyme-linked immunosorbent assay (ELISA) kit from IBL International in Tokyo, Japan, was used to detect the serum soluble α-Klotho concentration. Duplicate samples were analyzed, and the average of the two values was considered the final value. Rigorous quality control measures were implemented during the analysis of analyte concentrations on each ELISA plate.

### Covariables

This study considered potential confounding variables that could impact the relationship between the TyG index and the α-Klotho protein concentration. These covariables included sex, age, race/ethnicity, education level, marriage, smoking and drinking habits, body mass index (BMI), laboratory parameters such as serum uric acid, high-density lipoprotein cholesterol (HDL-C), and estimated glomerular filtration rate (eGFR), as well as comorbidities such as hypertension and chronic kidney disease (CKD). Race/ethnicity was classified into four categories: non-Hispanic white, non-Hispanic black, Mexican American, or other race. Marriages were classified as partnered (married, partnered) or other statuses (never married, separated, divorced, or widowed). Education levels were categorized as below high school, high school, and above high school. Smoking status was classified as never, former, or current based on definitions previously reported [23]. In terms of drinking status, a respondent was considered to be an alcohol drinker if they had consumed at least 12 drinks of alcohol per year in their lifetime [24]. BMI was calculated using the standard method based on weight and height, and eGFR was computed using the Chronic Kidney Disease Epidemiology Collaboration (CKD-EPI Cr) equation. Hypertension was diagnosed using antihypertensive medication, self-reported hypertension, or blood pressure measurements ≥ 140 (systolic)/90 (diastolic) mmHg [25]. CKD was diagnosed based on a urinary albumin-to-creatinine ratio (UACR) ≥ 30 mg/g and/or an eGFR < 60 mL/min/1.73 m^2^. For detailed information on the measurement techniques employed for these variables, please refer to www.cdc.gov/nchs/nhanes/.

### Statistical analysis

The statistical analyses were conducted following the guidelines outlined by the Centers for Disease Control and Prevention (CDC). Our analyses utilized a complex sampling design and sampling weights in accordance with NHANES analytical guidelines. Data for our study was obtained from household interviews, Mobile Examination Center (MEC) visits, and fasting samples during the NHANES survey. According to NHANES analytic guidelines on survey sample weights and their appropriate use, we should apply the Fasting Subsample 2 Year MEC Weight [21]. Sample weights were calculated as 1/5 × 2-year Fasting Subsample 2 Year MEC Weight for the years 2007–2016. Variables displaying a normal distribution are reported as the mean along with the standard deviation, while skewed variables are presented as the median with the interquartile range (25–75%). Proportions (%) were used to represent categorical variables. The t-test and Mann–Whitney U-test were used to compare normally and skewed distributed variables in continuous variables, respectively, while the χ2 test was utilized for comparing categorical data.

Multivariable linear regression models were utilized to examine the independent relationship between the TyG index and α-Klotho levels across three different models in both the groups with and without diabetes. In Model 1, no covariable were adjusted. Model 2 involved adjustments for sex, age, race, education level, and marriage. Model 3 included adjustments for all covariable in Model 2, as well as drinking status, smoking status, BMI, HDL-C, uric acid, eGFR, hypertension, and CKD. Weighted curve fitting was used to examine the association between the TyG index and α-Klotho levels. Stratified analyses were conducted based on sex, age, drinking status, and CKD in both groups to identify potential interactions.

Given that no a priori statistical power calculation was conducted, the sample size relied solely on the existing data. For the analysis, R software (version 4.2.1; R Foundation for Statistical Computing; http://www.Rproject.org), the R survey package (version 4.1–1), and free statistics software (version 1.7.1; Beijing Free Clinical Medical Technology Co., Ltd.) were utilized. Statistical significance was determined based on a two-sided *p* value < 0.05.

## Results

### Baseline characteristics of the study population

Among the 6439 subjects analyzed, 1577 individuals (24.5%) were identified as having diabetes mellitus. Table 1 presents a comparison of baseline demographic and clinical characteristics between participants with and without diabetes, representing approximately 112.77 million adults aged 40 years and older in the US. The average age, weighted for the sample, was 56.3 years, with women accounting for 52.9 percent of the total. The results indicated that, in comparison to those in the group without diabetes, a higher proportion of patients with diabetes were male, older, had a lower education level, and had a higher incidence of elevated BMI. Furthermore, the diabetes group demonstrated a higher incidence of comorbidities such as hypertension and CKD. Regarding laboratory measurements, individuals with diabetes had elevated uric acid levels, UACRs, and TyG levels but lower HDL-C levels, 25(OH)vitamin D levels, and eGFRs (*p* < 0.001). Notably, soluble α-Klotho concentrations were not significantly different between participants with and without diabetes (804.4 [659.2, 996.7] vs. 798.1 [657.2, 978.8] pg/mL; *p* = 0.071).

**Table 1:** Baseline characteristics of the study population according to diabetes mellitus status (*n* = 6439)

Variables	Total (*n* = 6439)	Without Diabetes (*n* = 4862)	With Diabetes (*n* = 1577)	*P* value
Sex, n (%)				0.0015
Male	3082 (47.1)	2263 (45.9)	819 (52.6)	
Female	3357 (52.9)	2599 (54.1)	758 (47.4)	
Age (years)	56.3 ± 10.4	55.4 ± 10.3	60.2 ± 9.9	< 0.001
Race/ethnicity, n (%)				< 0.001
Non-Hispanic white	1003 (6.5)	694 (5.8)	309 (9.7)	
Non-Hispanic black	779 (5.0)	558 (4.6)	221 (6.7)	
Hispanic	2828 (72.6)	2284 (74.8)	544 (62.9)	
Others	1829 (15.9)	1326 (14.8)	503 (20.7)	
Education level, n (%)				< 0.001
Below high school	866 (6.2)	574 (5.3)	292 (10.0)	
High school	2318 (31.6)	1688 (30.4)	630 (36.6)	
Above high school	3255 (62.2)	2600 (64.2)	655 (53.4)	
Marriage, n (%)				0.015
Partnered	4181 (71.1)	3197 (72.1)	984 (66.8)	
Others	2258 (28.9)	1665 (27.9)	593 (33.2)	
Smoking, n (%)				< 0.001
Never	3304 (52.2)	2532 (52.6)	772 (50.2)	
Former	2096 (33.1)	1521 (32.1)	575 (37.1)	
Current	1039 (14.7)	809 (15.3)	230 (12.7)	
BMI, kg/m^2^	29.6 ± 6.6	28.7 ± 6.1	33.1 ± 7.1	< 0.001
Drinking, n (%)				0.003
No	1323 (15.8)	963 (15.1)	360 (19.4)	
Yes	5116 (84.2)	3899 (84.9)	1217 (80.6)	
SBP, mmHg	124.2 ± 17.0	123.0 ± 16.6	130.0 ± 18.0	< 0.001
**Laboratory findings**				
HDL-C, mg/dl	55.2 ± 17.3	56.7 ± 11.1	48.4 ± 16.3	< 0.001
TC, mg/dl	201.2 ± 42.2	205.0 ± 41.0	184.4 ± 44.0	< 0.001
HbA1c, (%)	5.8 ± 1.0	5.5 ± 0.4	7.2 ± 1.6	< 0.001
Hemoglobin, g/dl	14.3 ± 1.4	14.3 ± 1.4	14.2 ± 1.5	0.030
Albumin, g/l	42.5 ± 3.0	42.6 ± 3.0	42.0 ± 3.3	< 0.001
Uric acid, mmol/l	329.0 ± 82.6	324.6 ± 80.2	348.2 ± 90.1	< 0.001
eGFR, ml/min	90.4 ± 17.5	91.2 ± 16.5	86.7 ± 21.0	< 0.001
25(OH)vitamin D, nmol/l, Median(IQR)	71.1 (53.7, 88.2)	72.8 (55.3, 89.3)	63.0 (46.4, 82.5)	< 0.001
UACR, mg/g, Median (IQR)	6.8 (4.6, 12.3)	6.3 (4.4, 10.8)	10.2 (5.8, 27.7)	< 0.001
FBG, mg/dL	110.0 ± 33.2	100.4 ± 9.6	153.0 ± 57.6	< 0.001
TyG index	8.7 ± 0.7	8.6 ± 0.6	9.2 ± 0.7	< 0.001
Fasting triglycerides, mg/dL, Median (IQR)	110.0 (77.0, 164.0)	106.0 (75.0, 154.0)	142.0 (95.0, 204.0)	< 0.001
α-Klotho, pg/ml, Median (IQR)	798.3 (657.5, 981.7)	798.1 (657.2, 978.8)	804.4(659.2, 996.7)	0.071
**Comorbidities**				
Hypertension, n (%)				< 0.001
No	3003 (51.7)	2583 (56.7)	420 (29.6)	
Yes	3436 (48.3)	2279 (43.3)	1157 (70.4)	
CKD, n (%)				< 0.001
No	5229 (86.0)	4210 (89.5)	1019 (70.5)	
Yes	1210 (14.0)	652 (10.5)	558 (29.5)	

*BMI* Body mass index, *SBP* Systolic blood pressure, *FBG* Fasting blood glucose, *TyG* Triglyceride glucose index, *eGFR* estimated glomerular filtration rate, *UACR* Urea albumin–creatinine ratio, *TC* Total cholesterol, *HDL-C* High-density lipoprotein cholesterol, *HbA1c* Glycosylated hemoglobin, *CKD* Chronic kidney disease

### The impact of the TyG index on the serum α-Klotho concentration

Univariable analysis, as detailed in Supplementary Table S1, revealed associations between the serum α-Klotho concentration and various demographic and clinical factors. These factors included age, sex, race/ethnicity, smoking status, alcohol consumption, the UACR, the eGFR, hypertension, CKD, and the TyG index. Notably, when the TyG index was treated as a continuous variable, a negative association with α-Klotho was evident (β value: -21.98 [-32.8, -11.17]). Additionally, a more detailed examination was conducted when the TyG index was categorized into tertiles. This analysis revealed that the α-Klotho level was significantly higher in the low-TyG subgroup compared to the intermediate-TyG subgroup (β value: 47.39 [29.45, 65.33]). However, no significant difference in α-Klotho expression was observed between the high-TyG group and the intermediate-TyG group.

### Associations between the TyG index and α-Klotho levels in individuals with or without diabetes

**Fig. 2:**
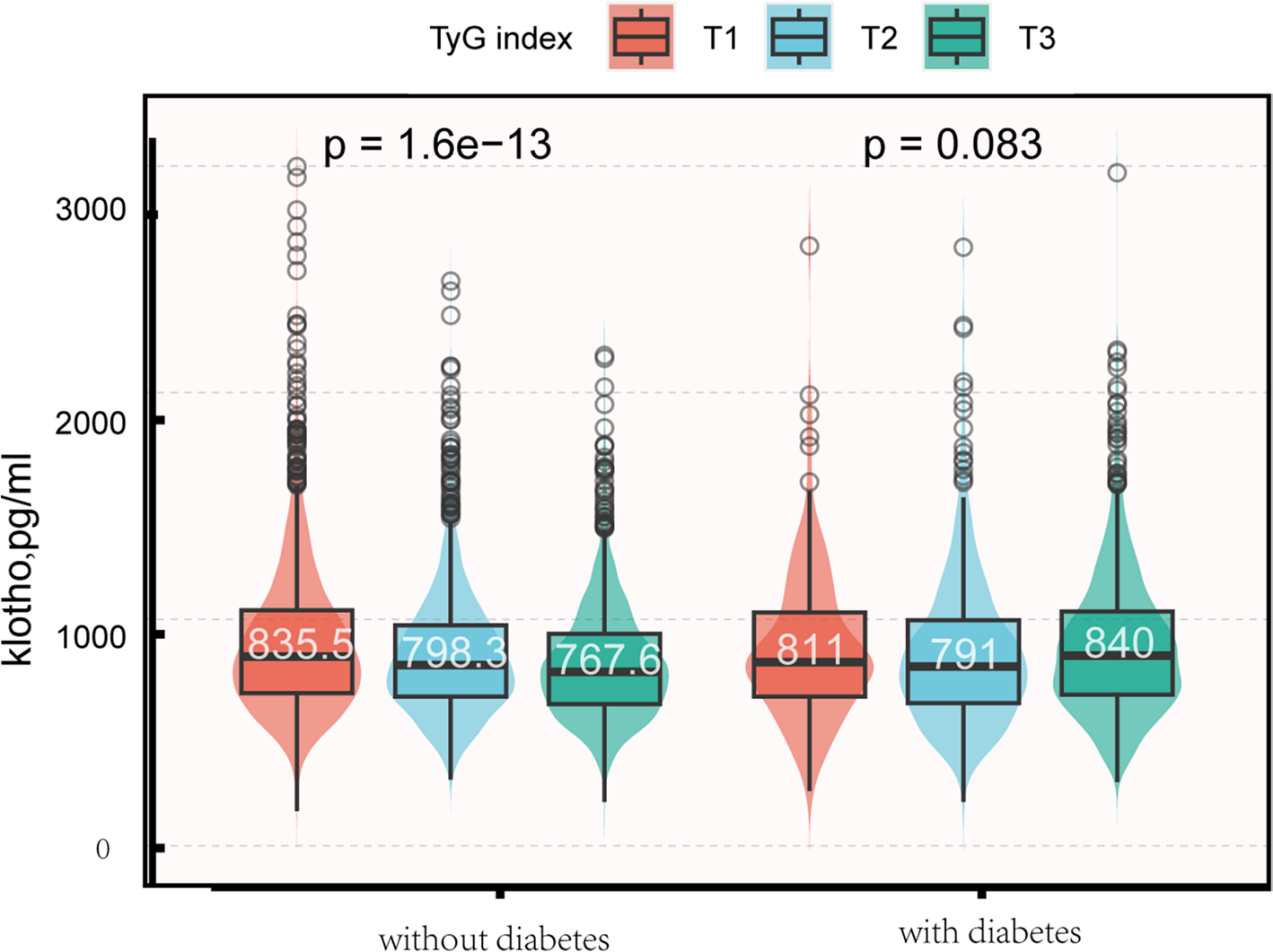
Distribution of soluble α-Klotho according to the TyG index according to diabetes status. TyG index T1: 6.94 to 8.32; T2: 8.33 to 8.81; T3: 8.82 to 12.41

Figure 2 shows the variation in α-Klotho concentration among the tertile TyG groups of participants with and without diabetes. In the group without diabetes, the low-TyG subgroup exhibited significantly higher α-Klotho levels compared to the high-TyG subgroup (835.5 vs. 767.6 pg/ml, *P* < 0.001). However, in participants with diabetes, α-Klotho levels were not significantly different among the three TyG syndrome groups (*p* = 0.082).

A subgroup analysis revealed that the presence of diabetes significantly influenced the relationship between the TyG index and the α-Klotho level (Supplementary Figure S1). To further explore the potential independent relationship between the TyG index and the α-Klotho concentration, a multivariable linear regression model was used. Researchers utilized both unadjusted and multivariable adjusted models to ensure the reliability of the findings. The criteria for selecting variables for adjustment were determined by three key considerations: (1) inclusion of variables that would lead to a minimum 10% change in the matched β upon addition to the model; (2) incorporation of variables exhibiting a significance level of *P* < 0.05 in the univariable linear regression analysis; or (3) inclusion of variables recognized as confounders based on literature and clinical judgment. Variables that showed significant multicollinearity were excluded.

**Fig. 3:**
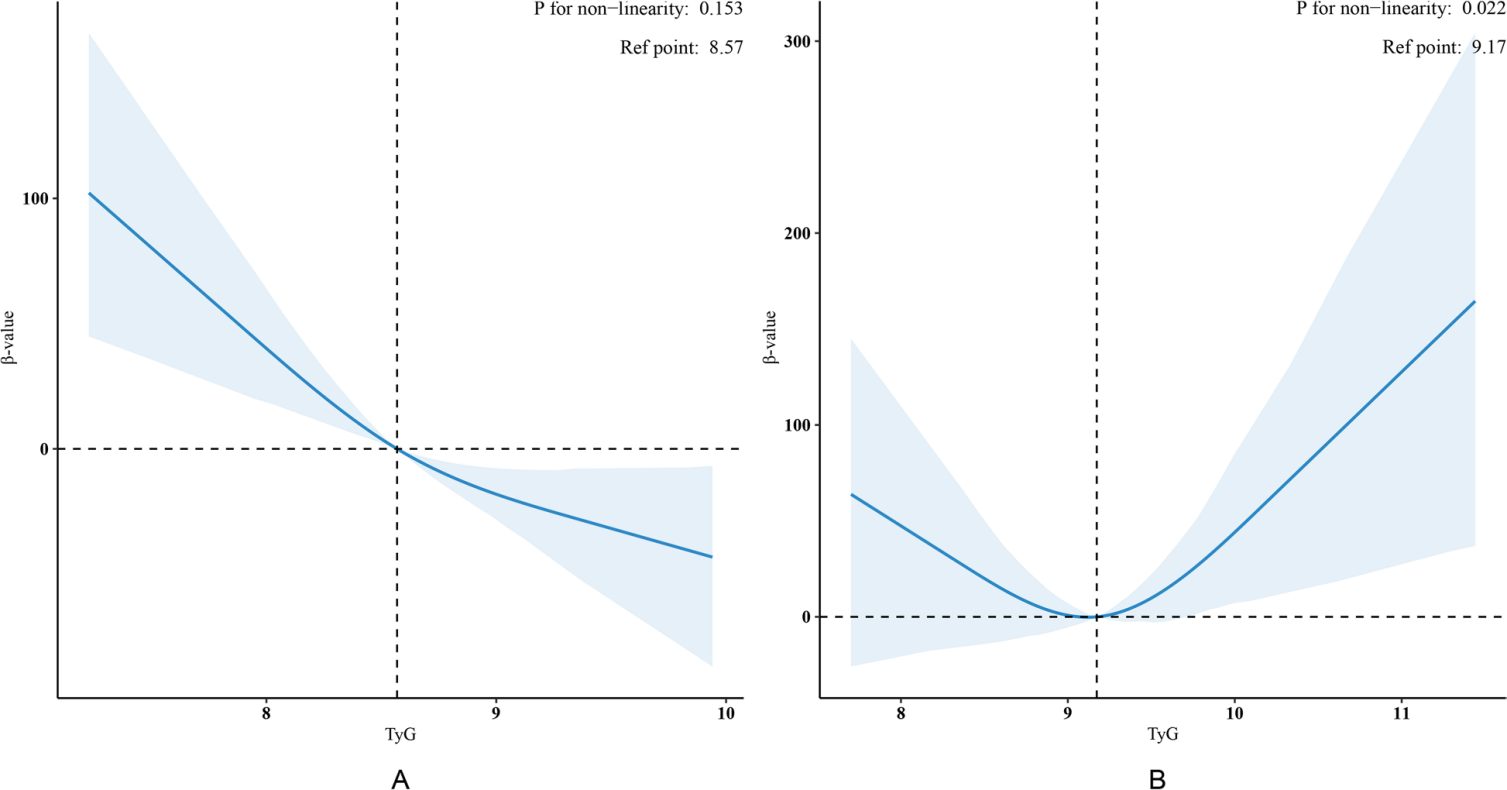
Associations between TyG and soluble α-Klotho levels in participants without diabetes (**A**) and with diabetes (**B**) according to survey-weighted multivariable linear regression based on restricted cubic splines. The results were adjusted for age, sex, race/ethnicity, marriage, education level, drinking status, smoking status, HDL-C, uric acid, eGFR, hypertension and CKD. HDL-C, high-density lipoprotein cholesterol; eGFR, estimated glomerular filtration rate; CKD, chronic kidney disease. Only 99% of the data is shown

After adjusting for age, sex, race, BMI, educational level, drinking status, smoking status, HDL-C, uric acid, eGFR, hypertension, and CKD, weighted multivariable regression analysis revealed that in the group without diabetes, the α-Klotho level decreased by 32.35 pg/ml (95% CI: -50.07, -14.64) for every one unit increase in TyG (*p* < 0.001). When the TyG index was transformed into a categorical variable, α-Klotho concentrations decreased by 29.6 pg/ml (95% CI: -59.42, 0.20) and 45.2 pg/ml (95% CI: -79.66, -10.76) in participants in the medium and high-TyG groups, respectively, compared with those in the low-TyG group (p for trend < 0.001 and p for trend = 0.011) (Table 2). Weighted curve fitting, after adjusting for all variables, revealed a linear correlation between the TyG index and α-Klotho levels (Fig. 3A).

**Table 2:** Associations between the TyG index and serum α-Klotho in middle-aged and older participants without diabetes according to the NHANES 2007–2016

Variable	Model I		Model II		Model III	
	β(95%CI)	*P* value	β(95%CI)	*P* value	β(95%CI)	*P* value
TyG index	-61.81 (-82.50, -41.13)	< 0.001	-49.40 (-69.24, -29.57)	< 0.001	-32.35( -50.07, -14.64)	< 0.001
subgroups						
T1	reference					
T2	-39.04 (-67.91, -10.16)	< 0.001	-30.92(-58.85, -2.99)	0.031	-29.6(-59.42, 0.20)	0.052
T3	-72.29 (-102.62, -41.95)	< 0.001	-57.92(-87.35, -28.49)	< 0.001	-45.2(-79.66, -10.76)	0.011
P for Trend		< 0.001		< 0.001		0.011

TyG tertiles: T1: 6.94 to 8.32; T2: 8.33 to 8.81; T3: 8.82 to 11.81

β is the effect size (pg/mL) of the change in the serum α-klotho concentration, and the 95% CI indicates the 95% confidence interval

Model I: adjusted for none

Model II: adjusted for age, sex, race/ethnicity, marriage, and education level

Model III: adjusted for age, sex, race/ethnicity, marriage, education level, body mass index, drinking status, smoking status, high-density lipoprotein cholesterol, uric acid, eGFR, hypertension, and CKD

*eGFR* estimated glomerular filtration rate, *CKD* Chronic kidney disease

In the group with diabetes, weighted curve fitting revealed a non-linear relationship between the TyG index and α-Klotho (Fig. 3B). Subsequently, TyG was triple-classified, and a multivariable regression analysis was conducted. The intermediate-TyG group served as a reference point. The analysis revealed that within the high-TyG group, for every one unit increase in TyG, the klotho level increased by 101.92 pg/ml (95% CI: 25.16, 178.68, *p* = 0.01). Conversely, no significant difference was observed in the low-TyG group (*p* = 0.262, Table 3). In the threshold effect analysis, the β value was 106.44 pg/ml (95% CI: 28.13, 184.74, *p* = 0.008) in participants with a TyG index ≥ 9.70 (Table S2).

**Table 3:** Associations between the TyG index and serum α-Klotho levels in middle-aged and older participants with diabetes according to the NHANES 2007–2016

Variable	Model I		Model II		Model III	
	β(95%CI)	*P* value	β(95%CI)	*P* value	β(95%CI)	*P* value
TyG index	24.95 (-9.16, 59.06)	0.149	22.54 (-11.39, 56.48)	0.189	22.54 (-11.39, 56.48)	0.189
subgroups						
T2	reference					
T1	31.6 (-14.88, 78.08)	0.18	25.99(-20.55, 72.52)	0.269	30.37(-23.24, 83.97)	0.262
T3	115.78 (37.13, 194.43)	0.004	100.81(24.93, 176.69)	0.01	101.92(25.16, 178.68)	0.01
P for Trend		0.005		0.012		0.008

TyG tertiles: T1 = 7.23 to 8.89; T2 = 8.90 to 9.46; T3 = 9.47 to 12.41

β is the effect size (pg/mL) of the change in soluble α-klotho level, and the 95% CI indicates the 95% confidence interval

Model I: adjusted for none

Model II: adjusted for age, sex, race/ethnicity, marriage, and education level

Model III: adjusted for age, sex, race/ethnicity, marriage, education level, body mass index, drinking status, smoking status, high-density lipoprotein cholesterol, uric acid, eGFR, hypertension, and CKD

*eGFR* estimated glomerular filtration rate, *CKD* Chronic kidney disease

### Stratified analyses based on sex, age, drinking status and CKD

**Fig. 4:**
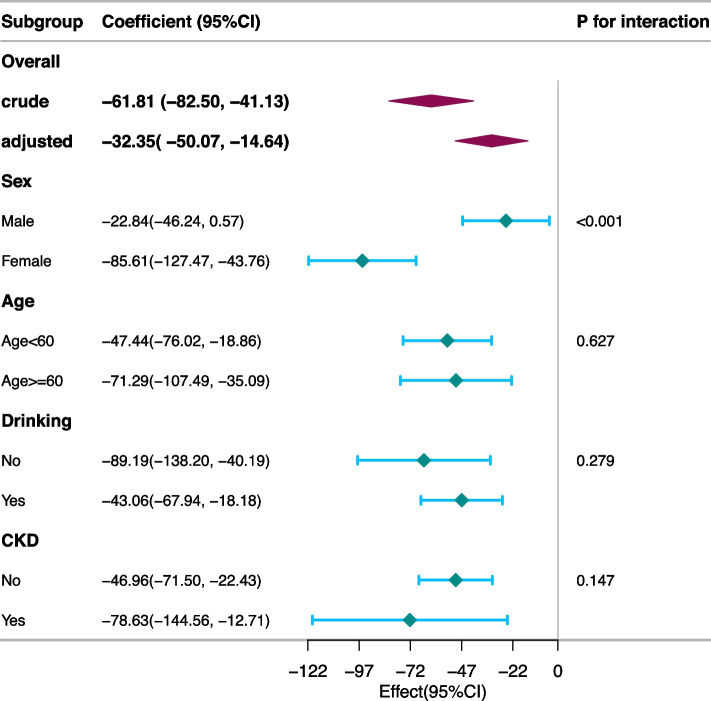
Survey-weighted stratification analysis of the association between the TyG index and soluble α-Klotho in participants without diabetes. CKD, chronic kidney disease

Figure 4 displays a forest plot illustrating the outcomes of subgroup analyses in participants without diabetes. Notably, the subgroup analysis revealed noteworthy sex-related differences in the TyG-Klotho association (p for interaction < 0.001). The association between the TyG index and α-Klotho was more pronounced in female participants than in male participants (β -85.61 [95% CI: -127.47, -43.76] vs. β -22.84 [95% CI: -46.24, 0.57]). Supplementary Figure S2 illustrates the consistency of the relationship between the TyG index and α-Klotho levels in the group with diabetes across subgroups stratified by sex, age, drinking status, and CKD (*p* values for interaction > 0.05).

## Discussion

In this study, we utilized data from the U.S. NHANES (2007–2016) for individuals aged 40 years and older. Our findings revealed a negative dose–response relationship between the TyG index and soluble α-Klotho concentration in individuals without diabetes. This relationship was particularly prominent among female patients. Among individuals with diabetes, high TyG levels were associated with elevated α-Klotho concentrations, but no sex differences were observed.

Numerous studies have explored the intricate relationship between IR and α-Klotho levels, but the results have been inconsistent. For instance, a study observed a reduction in plasma Klotho levels among individuals with type 2 diabetes and obesity [26]. Another observational study reported a negative association between Klotho and triglyceride levels in healthy adults [27]. These findings suggest that a decrease in Klotho may contribute to the development of glucose and lipid metabolism disorders. However, it has also been established that Klotho can induce IR through various mechanisms [7]. Our study revealed a negative correlation between the TyG index and the α-Klotho concentration in individuals without diabetes, consistent with the findings of previous research [6] indicating that a lower α-Klotho level is associated with more severe IR.

Some studies have shown that decreased Klotho levels are associated with inflammatory responses and oxidative stress [28]; conversely, heightened Klotho expression has been demonstrated to lower the levels of inflammatory factors and alleviate oxidative stress [29]. IR is widely recognized for its association with chronic inflammation, a condition arising from the presence of various proinflammatory cytokines and oxidative stress biomarkers. Specifically, interleukin-1 beta, interleukin-6, and adipocytokines have been identified as pivotal contributors to this inflammatory response [30, 31]. Prolonged exposure to elevated levels of these proinflammatory biomarkers triggers the activation of cytokine signaling pathways, which in turn leads to the ultimate impairment of insulin signaling receptor activation in β-cells located within the pancreatic islets. Furthermore, oxidative stress detrimentally affects the functionality of pancreatic β-cells, leading to apoptosis and further exacerbating insulin resistance [32].

Endothelial dysfunction is intimately associated with insulin resistance. Previous investigations have suggested the involvement of the miR-21 and MARK/ET-1 pathways in the development of endothelial dysfunction induced by pathological hyperinsulinemia and hyperglycemia [33]. Conversely, Klotho proteins have been demonstrated to alleviate endothelial dysfunction by inhibiting the ROS/p38MAPK and downstream NF-κB signaling pathways [34]. Both the TyG index and the Klotho level have been shown to be related to muscle mass. An analysis of 9,477 participants aged 40 years and older in the Korean National Health and Nutrition Examination Survey revealed a association between elevated TyG scores and diminished muscle mass [15]. Moreover, investigations have revealed a significant reduction in muscle mass in mice lacking the klotho protein [35], as well as a positive correlation between klotho levels and muscle mass in individuals with chronic obstructive pulmonary disease [36]. The TyG index influences serum klotho expression through various pathways, including inflammatory factor expression, oxidative stress, endothelial function, and muscle mass. Nonetheless, establishing a definitive causal relationship between these factors remains a complex challenge.

The decrease in soluble α-Klotho levels has been reported in individuals with diabetes [37, 38]. However, our findings did not reveal a significant difference in α-Klotho concentrations between groups with and without diabetes. The reason for this difference remains unclear. Our study also revealed that, unlike individuals without diabetes, in patients with diabetes, the TyG index and the α-Klotho level were positively correlated. Furthermore, subgroup analysis indicated that there was no significant interaction effect between sex, age, drinking, or CKD on the association between the TyG index and the α-Klotho concentration, confirming the stability of the results.

Research has consistently indicated that α-Klotho plays a role in both inducing IR [7, 39] and modulating the peripheral response to insulin. Notably, Klotho knockout mice exhibit increased insulin sensitivity, while Klotho transgenic mice exhibit IR [6]. A significant positive correlation was observed between hyperglycemia and the concentration of α-Klotho. It has been suggested that the α-Klotho protein may promote IR as a protective mechanism against the adverse effects of lipotoxicity and apoptosis in individuals who are overfed [40]. Klotho disrupts insulin-induced phosphorylation, impedes glucose uptake stimulated by insulin, and decreases the level of malonyl CoA. Consequently, this process encourages the oxidation of fatty acids, reduces the accumulation of lipids inside cells, increases the threshold for apoptosis, and extends the lifespan of cells [39]. These findings align with our observations in patients with diabetes, where individuals with elevated TyG levels demonstrated increased α-Klotho levels.

Klotho-mediated insulin resistance may function as a mechanism to counteract the aging process [41]. Moreover, reduced insulin-like peptide signaling has been shown to extend the lifespan of nematodes, flies, and rodents [42]. However, an excessively elevated level of α-Klotho in patients with diabetes may indicate a compensatory reaction to stress and inflammation, thus potentially being linked to an unfavorable prognosis. Several studies have suggested that significantly high levels of α-Klotho are associated with increased mortality [43, 44].

According to the sex-stratified analysis of this study, the TyG index demonstrated a more robust correlation with α-Klotho in female participants without diabetes. Previous research has shown sex differences in the relationship between triglyceride metabolism and α-Klotho [45]. The underlying mechanisms for this distinction are not fully understood and may stem from variances in hormonal pathways between the sexes [46, 47]. Additionally, studies have revealed that female α-Klotho levels are strongly associated with conditions such as depression and obesity [48, 49], in which estrogen may play a significant role [50].

This study has several key strengths. First, the large sample size and representativeness of the participants contributed to improving the statistical power and generalizability of the research results. Second, the data analysis included comprehensive adjustments for potential confounding factors, which helps to reduce interference from other variables in the relationship between the main variables. Additionally, this study revealed, for the first time, a dose–response relationship between TyG and α-Klotho in both participants with and without diabetes. Finally, the sex stratification analysis revealed a significant sex interaction, emphasizing the crucial role that sex may play in the relationship between these two variables. These strengths collectively enhance the credibility and provide a deeper understanding of the findings.

### Limitations

In our study, we explored the complex relationship between the TyG index and the level of soluble α-Klotho in a nationally representative population. However, it is crucial to acknowledge some limitations that should be considered when interpreting our findings. First, using stored excess serum for measuring serum α-Klotho could introduce measurement bias, which may affect the quality of the samples. Second, the self-reported nature of the sociodemographic factors and comorbidity status of the participants may have influenced the accuracy of the measurements. Finally, despite our diligent efforts to account for multiple potential confounding factors, the possibility of residual confounding persists, which could affect the validity of our results. Furthermore, the cross-sectional study design employed in our research does not permit the establishment of a causal relationship between TyG and soluble α-Klotho.

## Conclusion

Among individuals without diabetes, the study revealed a negative association between the TyG index and soluble α-Klotho, exhibiting a more pronounced effect, particularly among female participants. Furthermore, the relationship between the TyG index and soluble α-Klotho may be affected by the presence or absence of diabetes. To gain a more comprehensive understanding of the relationship between IR and α-Klotho levels, further research is essential.

### Supplementary Information

Supplementary Material 1: Table S1. Correlation analysis of potential factors related to the soluble α-Klotho level.

Supplementary Material 2: Figure S1. Survey-weighted subgroup analysis of the TyG index and soluble α-Klotho level based on diabetes mellitus.

Supplementary Material 3: Table S2. The non-linear relationship between the TyG index and soluble α-Klotho in middle-aged and older participants with diabetes.

Supplementary Material 4: Figure S2. Survey-weighted stratification analysis on the association between TyG index and α-Klotho in participants with diabetes.

## Data Availability

Data is provided within the manuscript or supplementary information files.

## Abbreviations

IR: Insulin resistance
TyG: Triglyceride-glucose
CI: Confidence interval
NHANES: The National Health and Nutrition Examination Survey
HbA1c: Glycosylated hemoglobin
FBG: Fasting blood glucose
TG: Fasting triglyceride
BMI: Body mass index
HDL-C: High-density lipoprotein cholesterol
eGFR: Estimated glomerular filtration rate
CKD: Chronic kidney disease
UACR: Urine albumin-to-creatinine ratio
