# Supplementary material for: Associations between the TyG index and the ɑ-Klotho protein in middle-aged and older population relevant to diabetes mellitus in NHANES 2007–2016
Source: Lipids Health Dis. 2024 Jun 21;23:188. doi: 10.1186/s12944-024-02172-3 (PMC11191244; doi:10.1186/s12944-024-02172-3)
Supplement: Supplementary file 1 — Supplementary Material 1: Table S1. Correlation analysis of potential factors related to the soluble α-Klotho level. [file 12944_2024_2172_MOESM1_ESM.docx]

**Table S1. Correlation analysis of potential factors related to the soluble α-Klotho concentration**

| Variables | β (95%CI) | P value |
| --- | --- | --- |
| Sex |  |  |
| Male | Ref. |  |
| Female | 60.87 (46.24,75.49) | < 0.001 |
| Age，years | -1.67 (-2.34, -0.99) | < 0.001 |
| Race/ethnicity |  |  |
| Non-Hispanic white | Ref. |  |
| Non-Hispanic black | 23.81 (-4.2,51.81) | 0.096 |
| Hispanic | -22.55 (-44.1, -1) | 0.04 |
| Others | 47.17 (24.13,70.21) | < 0.001 |
| Education level, n (%) |  |  |
| Below high school | Ref. |  |
| High school | -11.46 (-34.91,12) | 0.338 |
| Above high school | 12.56 (-9.95,35.08) | 0.274 |
| Marriage |  |  |
| Partnered vs others | 6.84 (-8.55,22.23) | 0.384 |
| BMI,kg/m^2^ | -0.37 (-1.49,0.74) | 0.511 |
| Drinking,n (%) | -68.7 (-86.8, -50.6) | < 0.001 |
| Smoking status, n(%) |  |  |
| Never | Ref. |  |
| Former | -65.53 (-81.88, -49.18) | < 0.001 |
| Current | -74.2 (-95.02, -53.38) | < 0.001 |
| HDL-C,mg/dl | 0.48 (0.04,0.92) | 0.031 |
| HbA1c,(%) | -11.22 (-33.71,11.26) | 0.328 |
| eGFR,ml/min | 1.4 (1.02,1.78) | < 0.001 |
| 25(OH)vitamin D,nmol/l | -0.32 (-0.59, -0.06) | 0.017 |
| UACR,mg/g | -0.01 (-0.03,0.01) | 0.158 |
| Diabetes, n(%) | 19.7 (2.63,36.77) | 0.024 |
| Hypertension,n(%) | -8.07 (-22.79,6.64) | 0.282 |
| CKD,n (%) | -25.47 (-44.26, -6.68) | 0.008 |
| TyG index | -21.98 (-32.8, -11.17) | < 0.001 |
| TyG index groups |  |  |
| T1 | 47.39 (29.45,65.33) | < 0.001 |
| T2 | Ref. |  |
| T3 | -3.62 (-21.56,14.31) | 0.692 |

BMI, body mass index; HDL-C, high-density lipoprotein cholesterol; HbA1c, glycosylated hemoglobin; eGFR, estimated glomerular filtration rate; UACR, urea albumin–creatinine ratio; CKD, chronic kidney disease; TyG, triglyceride glucose index.
