# Supplementary figures and images for: Associations between the TyG index and the ɑ-Klotho protein in middle-aged and older population relevant to diabetes mellitus in NHANES 2007–2016
Source: Lipids Health Dis. 2024 Jun 21;23:188. doi: 10.1186/s12944-024-02172-3 (PMC11191244; doi:10.1186/s12944-024-02172-3)

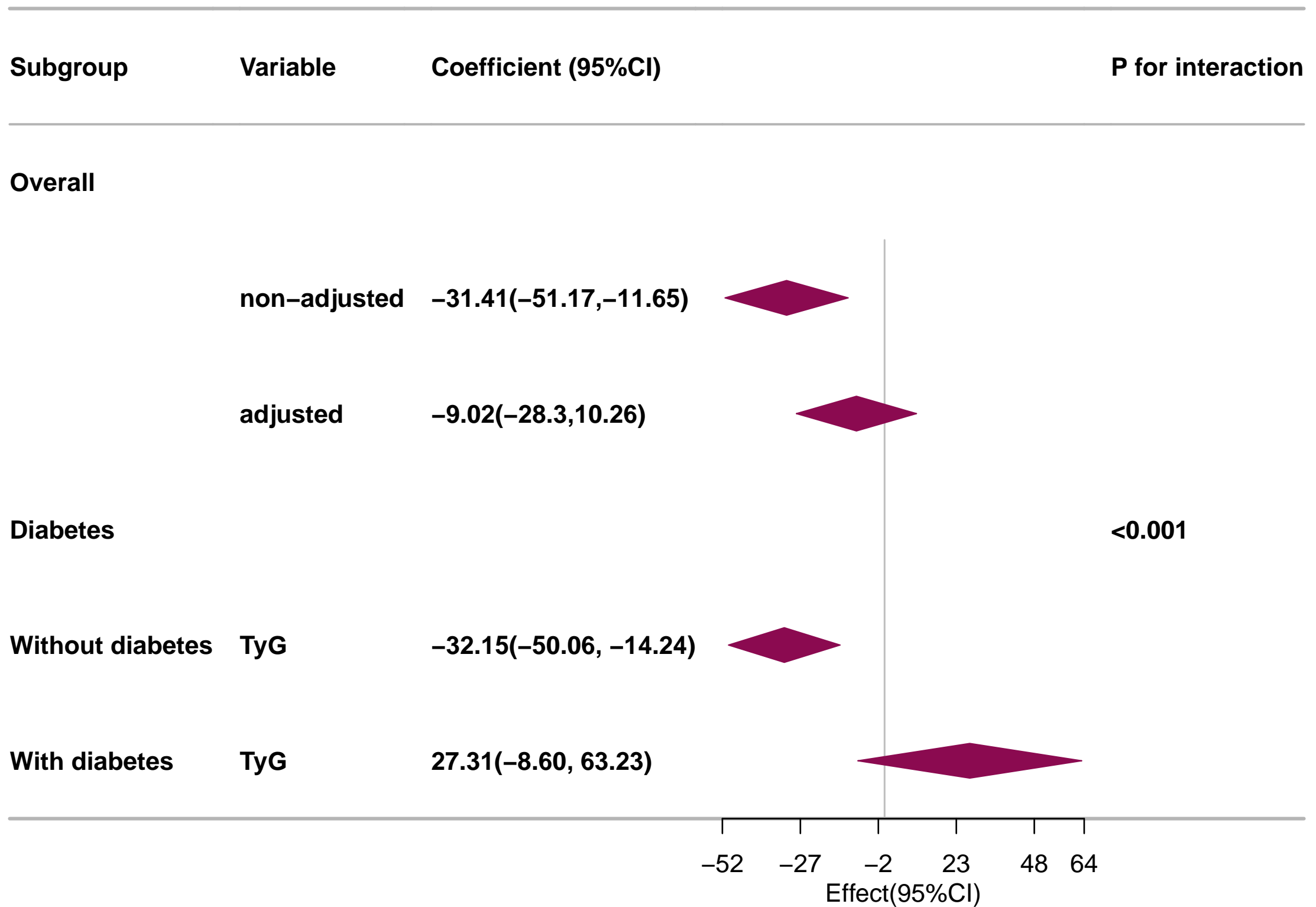

Supplement: Supplementary file 2 — Supplementary Material 2: Figure S1. Survey-weighted subgroup analysis of the TyG index and soluble α-Klotho level based on diabetes mellitus. [file 12944_2024_2172_MOESM2_ESM.pdf]

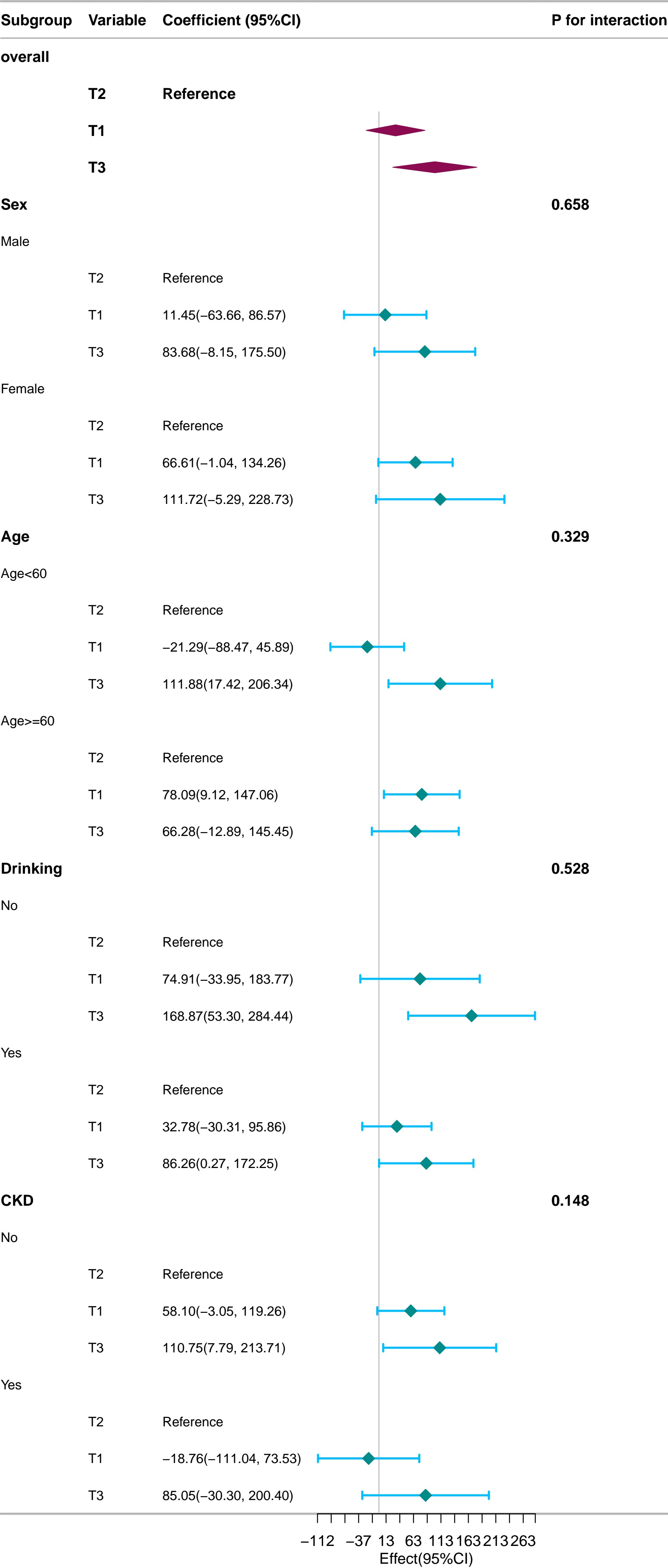

Supplement: Supplementary file 4 — Supplementary Material 4: Figure S2. Survey-weighted stratification analysis on the association between TyG index and α-Klotho in participants with diabetes. [file 12944_2024_2172_MOESM4_ESM.pdf]
