# Supplementary material for: Associations between the TyG index and the ɑ-Klotho protein in middle-aged and older population relevant to diabetes mellitus in NHANES 2007–2016
Source: Lipids Health Dis. 2024 Jun 21;23:188. doi: 10.1186/s12944-024-02172-3 (PMC11191244; doi:10.1186/s12944-024-02172-3)
Supplement: Supplementary file 3 — Supplementary Material 3: Table S2. The non-linear relationship between the TyG index and soluble α-Klotho in middle-aged and older participants with diabetes. [file 12944_2024_2172_MOESM3_ESM.docx]

**Table S2 The non-linear relationship between the TyG index and α-Klotho in middle-aged and older participants with diabetes.**

| Threshold of TyG | β-value | 95% CI | P-value |
| --- | --- | --- | --- |
| <9.70 | -26.72 | (--64.77,11.32) | 0.168 |
| ≥9.70 | 106.44 | (28.13,184.74) | 0.008 |
| Likelihood Ratio test |  |  | < 0.001 |

β is the effect size (pg/mL) of the change in soluble α-klotho level, and the 95% CI indicates the 95% confidence interval.

Adjusted for age, sex, race/ethnicity, marriage, education level, body mass index，drinking status，smoking status, high-density lipoprotein cholesterol, uric acid, eGFR, hypertension and CKD.

eGFR, estimated glomerular filtration rate; CKD, chronic kidney disease.
